# The Surgical Treatment of Pelvic Bone Metastases

**DOI:** 10.1155/2015/525363

**Published:** 2015-02-24

**Authors:** Daniel A. Müller, Rodolfo Capanna

**Affiliations:** ^1^Department of Orthopedic Surgery, Balgrist University Hospital, 8008 Zurich, Switzerland; ^2^Department of Orthopedic Oncology, Careggi University Hospital, 50139 Florence, Italy

## Abstract

Pelvic bone metastases are a growing concern in the field of orthopedic surgery. Patients with pelvic metastasis are individually different with different needs of treatment in order to attain the best possible quality of life despite the advanced stage of disease. A holistic collaboration among the oncologist, radiation therapist, and orthopedic surgeon is mandatory. Special attention has to be directed to osteolytic lesions in the periacetabular region as they can provoke pathological fractures and subsequent functional impairment. Different reconstruction techniques for the pelvis are available; the choice depends on the patient's prognosis, size of the bone defect, and response of the tumor to adjuvant treatment. If all the conservative treatments are exhausted and the patient is not eligible for surgery, one of the various percutaneous ablation procedures can be considered. We propose a pelvic analogue to the treatment algorithm in long bone metastasis and a scoring system in pelvic metastasis. This algorithm aims to simplify the teamwork and to avoid under- or overtreatment of pelvic bone metastases.

## 1. Introduction

Primary cancer can spread via the blood or lymphatic circulation to distant organs and form a metastasis. In theory, any organ of the body can be affected, but after lung and liver, bone is the third most common site for metastases. Prostate (32%), breast (22%), kidney (16%), lung and thyroid cancer have a high risk for metastatic bone disease. In fact, these primary carcinomas account for 80% of all the metastases to the bone [1].

Metastatic lesions are found most frequently in the spine, followed by the pelvis. Indeed 833 (18.8%) of all 4431 metastatic lesions registered in the archive of the Rizzoli institute [2] were found to occur in the pelvic region: 559 (12.6%) are located in the ilium, 80 (1.8%) in the ischium, and 53 (1.2%) in the pubis.

In most of the cases complete cure of the disease is not possible and treatment is aimed at palliation. Nevertheless, metastatic carcinoma to the pelvis and the acetabulum decreases seriously the quality of life of the patient and necessitates further treatment. Surgical intervention helps to achieve adequate pain control and to prevent or stabilize pathological fractures. However, in selected cases, complete resection may improve the survival rate of the patient.

The overall prognosis of patients with bone metastasis is extremely variable depending on the site of the lesion, type of primary carcinoma, and existence of further metastasis.

In the past decades, the life expectancy of patients with metastatic carcinoma has improved considerably because of advances in chemotherapy, immunotherapy, hormonal treatment, and radiotherapy [3]. However, this has resulted in an increase in number of patients at risk of developing bone metastases or experiencing a pathological fracture [4]. These patients demand a more reliable and stable reconstructive technique.

Myeloma and lymphoma bone lesions have been shown to have a similar biological behavior as metastatic bone disease and the mechanical implications are comparable. However, chemotherapy and radiotherapy are still the cornerstones of treatment for all lymphomas. In lymphoma patients, bone lesions at risk for a fracture are often successfully treated with chemotherapy and radiotherapy in combination with rest and non-weight-bearing. Surgery is only indicated in pathological fractures with major functional impairments, whereas the timing remains a controversial issue [5]. If fracture location and patient condition allow, the surgical treatment can even be delayed until chemotherapy and radiation therapy are finished [5]. In summary, surgical treatment of primary bone lymphoma should aim to restore function and pain while minimizing potential delay in chemotherapy initiation.

To date, there is no officially accepted treatment algorithm for pelvic bone metastasis. Orthopedic surgeons, oncologists, or radiotherapists have been treating pelvic metastasis without any guidelines to consider the indications for surgical treatment. The following overview discusses the different possible surgical techniques and their indications and limitations in dealing with pelvic bone metastasis. The chosen procedure should offer an adequate treatment to the patient to achieve the best possible quality of life while avoiding under- or overtreatment.

## 2. Anatomic Regions of the Pelvis

Metastatic lesions affect the strength of bone reducing stress transmission and the ability to absorb energy. The evaluation of the risk of fracture in a metastasis of the pelvis is guided by its appearance and its location.

Osteolytic lesions are more at risk of fracture than osteoblastic or mixed lesions. Those with a permeative pattern of osteolysis have the same risk of fracture as the more classic types, which show a discrete area of lysis. Permeative osteolysis may be underestimated on plain radiographs, but MRI usually reveals the real extent of the disease.

**Figure 1:**
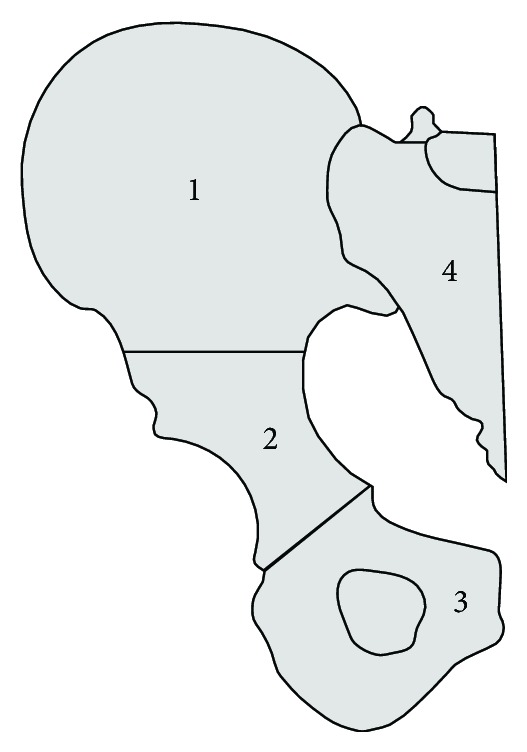
Anatomic regions of the pelvis according to the Enneking classification.

Highly stressed anatomical sites are particularly predisposed for pathological fractures. According to the Enneking classification [6] the pelvic girdle is divided into 4 different regions as shown in Figure 1.

Zones 1 and 3 are comparable to non-weight-bearing and expendable bones of the extremity and trunk (clavicle, sternum, and fibula). Zone 2 equates to the articular part of major long bones (humerus, femur, and tibia).

The periacetabular (zone 2) lesions are at greater risk for mechanical failure with progressive destruction of the hip joint. Metastatic lesions in zones 1 and 3, even if they are osteolytic, do not compromise the mechanical stability of the pelvic ring.

## 3. Patient Classification

The multidisciplinary approach to bone metastasis needs a good functioning interaction between orthopedic surgeon, oncologist, and radiotherapist, especially when surgery is needed. Capanna and Campanacci [7] introduced in 2001 an algorithm in long bone metastases providing an easy tool for all involved specialists to find an adequate treatment. The patients are divided into 4 classes: (1) solitary lesion with good prognosis; (2) pathologic fracture; (3) impending fracture; (4) other lesions (see Table 1). In selecting the adequate treatment in long bones and pelvis, important parameters as expected survival, the type and stage of the tumor, visceral spread, the time interval from the primary lesion, the risk of pathological fracture, and the sensitivity to chemotherapy, hormone therapy, and irradiation are considered.

## 4. Guidelines for Multidisciplinary Treatment

**Figure 2:**
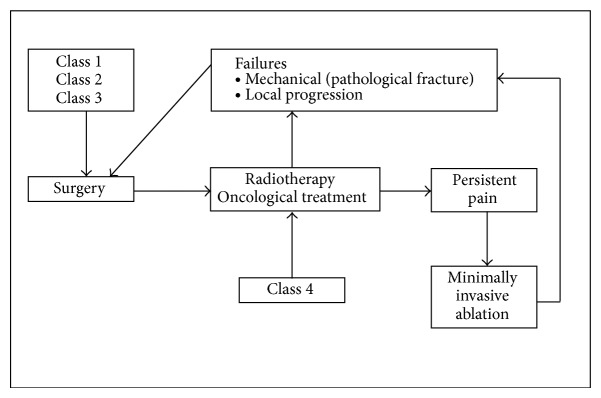
Indications for surgical and conservative treatment according to the patient classes.

The treatment depends on the patient's prognosis (Capanna Classes 1–4), the exact site of the metastasis in the pelvis (Enneking zones 1–3), and the amount of bone loss of the periacetabular region. A schematic overview of a proposed treatment algorithm is given in Figure 2.

All patients in Classes 1, 2, and 3 should have priority referral to an orthopedic oncologist for surgical treatment. After the operation they will be sent back to an oncologist and a radiotherapist for the evaluation of adjuvant treatment. Patients in Class 4 are treated first conservatively by chemotherapy, hormone therapy, and/or radiation therapy.

## 5. Patient Class 1

Class 1 includes those patients with a single metastatic lesion of a primary tumor with a good prognosis and an interval of more than three years from detection of the primary lesion to the development of bone metastasis. Primary tumors with a favorable prognosis include well-differentiated thyroid, prostate, breast, when sensitive to hormonal treatment or chemotherapy, clear-cell renal, and colorectal carcinoma. The metastasis is treated as a primary tumor and the operation aims to achieve a long-term cure, both oncological and mechanical. Previous studies have reported that curettage of single metastatic lesions in the pelvis is associated with high mortality and decreased survival compared with wide resection and have justified consideration of a radical surgical approach to achieve tumor control [8–14]. Surgery is probably one of the most important aspects of multimodal strategy in patients with few metastases when a curative attempt is made possible. Despite the emergence of molecular targeted systemic therapy (angiogenesis inhibitors for metastatic renal cell carcinoma) cure is uncommonly achieved in the absence of surgical resection [15].

The Enneking zones 1 and 3 do not require any reconstruction following the tumor resection because the ambulation capability is still preserved. A reinforcement by synthetic mesh helps to avoid visceral herniation. Relatively thin skin, diminished subcutaneous tissue, and lack of muscle mass overlying the anterior part of the pelvis increase the risk for skin necrosis and wound complications [1].

**Figure 3:**
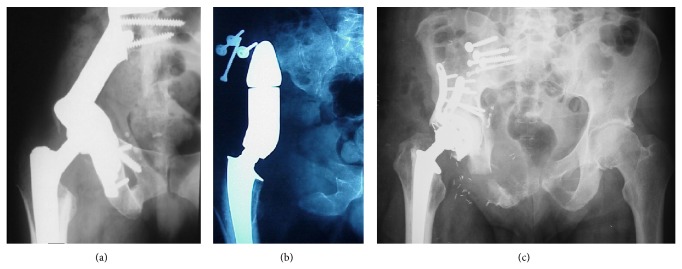
Different reconstruction techniques after wide resection of a periacetabular lesion. (a) Megaprosthesis; (b) saddle prosthesis; (c) massive allograft with total hip replacement.

Resections in zone 2 alone or in combination with the adjacent regions necessitate further reconstruction to prevent disability and gait disturbances. The periacetabular region can be replaced by custom-made or modular megaprosthesis, saddle prosthesis, or massive allograft in combination with a total hip replacement (Figure 3).

The saddle prosthesis (Link, Hamburg, Germany) was designed by Nieder et al. [16] for large acetabular defects in revision hip arthroplasty. Since the 1908s the saddle prosthesis was also used for reconstruction after periacetabular tumor resection. A notch has to be created in the iliac remnant, in some cases with an allograft augmentation providing more stability. The saddle articulates with the iliac notch and does not require an exact anatomic fit. A high risk of complications (ranging from 33 to 65%) are reported [12, 17, 18]. The major complications consist of wound problems (18–37%), transient peroneal nerve paresis, and neuropraxia of the sciatic and femoral nerves because of the manipulation of the femur and fractures of the remaining iliac wing (0–7%), which generate leg length discrepancies and dislocations (0–18%). The long-term functional outcome was poor with limited hip flexion [18].

Therefore the saddle prosthesis cannot be recommended for reconstruction after periacetabular tumor resections and remains a salvage procedure for extreme cases.

Custom-made and modular megaprostheses are useful in cases in which standard components are not sufficient. Intraoperative still modifiable modular components (especially the anteversion of the femoral neck) help to achieve better stability of the hip joint. The few available data in the literature indicates a satisfying functional outcome: most of the reported patients can ambulate without pain [19].

Wide tumor resections in the pelvis are reported to have a high rate of complications. But skeletal metastases decrease the quality of life, especially the loss of mobility, independence, and social functioning of a patient [20]. In spite of the mentioned complications, the surgical treatment of pelvic metastasis improves significantly the quality of life and morbidity of the patients [21, 22]. The decision to expose the patient to the burden of major surgery should be well considered and restricted to patients with a good prognosis and major functional impairments.

## 6. Patient Classes 2 and 3

Impending and pathological fractures necessitating a surgical intervention are all located in the periacetabular region (Enneking zone 2). The principle goal of the surgical treatment is to prevent a pathological fracture (Class 3) or to restore the mechanical integrity and function (in particular ambulation) if the fracture already occurred (Class 2). Preoperative angiography and selective embolization are recommended in highly vascular lesions such as clear-cell renal or thyroid carcinoma. If wide oncological margins are achieved at resection, postoperative radiotherapy can be avoided, but it is still recommended after marginal or intralesional procedures or in patients presenting with a pathological fracture. It should be delivered with full doses (3000 to 5000 cGy) and not with levels used for palliative control of pain.

**Figure 4:**
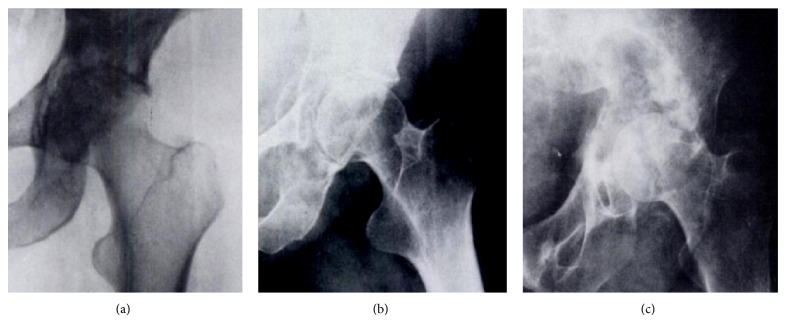
Classification of acetabular defects according to Harrington. (a) Integrity of medial and superior periacetabular bone (Group I). (b) Medial wall insufficiency (Group II). (c) Medial wall and supra-acetabular destruction (Group III). Group IV (no image): total collapse of acetabulum.

The amount of the periacetabular bone loss dictates the type of surgery. A good tool to indicate the acetabular destruction is the Harrington Classification, ranging from Groups I to IV (Figure 4). 

*Harrington Group I.* If the subchondral bone of the acetabulum is still intact, a simple curettage of the lesion may be performed with cement filling, thus preserving the hip. This procedure may be even carried out percutaneously. Metal pins or bars inserted into intact bone may be used as augmentation to reinforce the acetabular dome. Often the normal congruity of the acetabular surface shows already a disruption. But the unaffected periacetabular bone is still sufficient for a cemented conventional hip prosthesis. The incidence of loosening and migration will not exceed the amount seen in routine total hip replacement. Porous-coated implants have the disadvantage of requiring bone ingrowth for stability. These implants should not be used as the ingrowth is impaired by the cancer and chemo- or radiation therapy applied postoperatively. 

**Figure 5:**
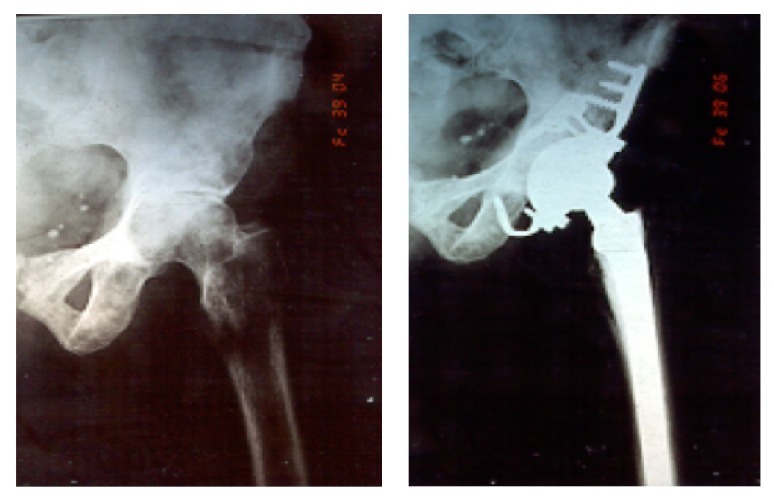
Pre- and postoperative radiographies for Harrington Class 2 bone defect.

*Harrington Group II.* The medial wall of the acetabulum is destroyed but the superior part (roof) and the lateral wall is still preserved. The use of a conventional prosthesis would lead to a medial migration and consequent loosening. Therefore the cup has to be fixed with the help of a reinforcement ring; otherwise the reconstruction will fail (Figure 5). 

*Harrington Group III. *Extensive osteolysis affects not only the medial wall but also the roof and the lateral rim of the acetabulum. In most patients also the inferior part is functionally nonexistent. So there is no possibility to adequately fixate a conventional cup or a reinforcement ring. Reconstruction typically involves using an implant and cement with internal fixation that extends into uninvolved portions of the pelvis. The large defect has to be replaced first by cement or an allograft to allow an implantation of a cup component afterwards. The use of cement is preferable as the addition of antibiotic drugs lowers the infection risk and an immediate weight-bearing of the affected limb is possible. The disease of the host bone and the irradiation therapy impair the union and ingrowth of an allograft postoperatively. Pseudarthrosis, allograft fractures, prolonged functional impairments, and a higher infection rates are resulting.

**Figure 6:**
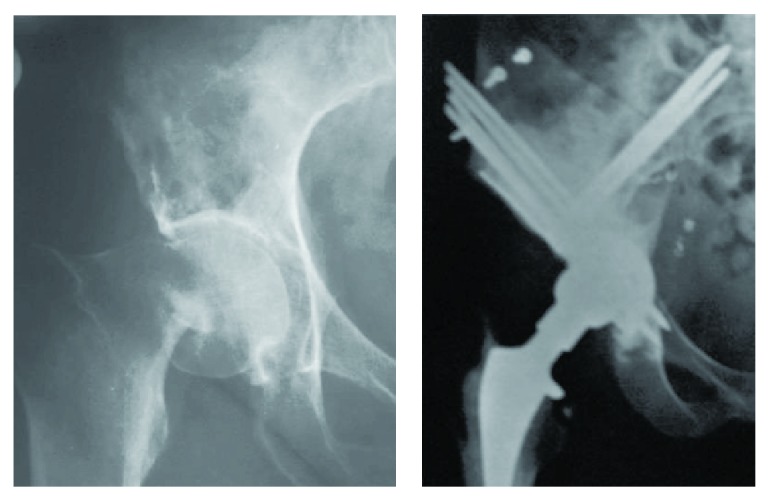
Pre- and postoperative radiographies of Class 3 acetabular defect using the Harrington technique for reconstruction.

Harrington described a technique [23] using large threaded pins placed within the surrounding hemipelvis to support the cemented acetabular component. These pins are used to transform the weight-bearing stresses from the component placed in the deficient acetabular bone to the unaffected bone of the remaining pelvis (Figure 6). This technique is challenging and requires an understanding of the pelvic anatomy and spatial orientation. Placing a finger in the sciatic notch while drilling helps to orientate the anteroposterior pins and protects the sciatic nerve.

Every effort must be made to improve stability of the hip and to avoid subsequent dislocation. Intrinsically stable joints should be implanted if possible. The use of either a snap-fit socket or a large prosthetic femoral head (size 28 to 32 mm) is strongly recommended. 

*Harrington Group IV.* The acetabulum is collapsed completely and can be restored only through a resection. The used reconstructions techniques are the same as in Class 1 patients except that aiming for wide margins is not necessary.

A summary of the different reconstruction techniques and their indications is shown in Table 2.

## 7. Suggestion for Surgical Treatment in Classes 2 and 3

Concordant to metastatic lesions in long bones [7] an adapted scoring system is introduced for assessment of periacetabular metastases in patients belonging to Class 2 or 3 (Table 3). Additionally to the size of defect, the expected survival (Table 4) and the response of the tumor to adjuvant treatment (Table 5) were taken into account. The aim of the scoring system is selecting the most appropriate surgery to avoid over- or undertreatment of the metastatic lesions. The surgical treatment should be more aggressive in an expected long survival of the patient and big lesions, which do not improve during adjuvant therapies.

## 8. Patients Class 4

This class includes patients with multiple osteoblastic lesions at any site and osteolytic or mixed lesions in non-weight-bearing bones (Enneking zones 1 and 3) who do not match the criteria for Class 1. Patients in Class 4 should be treated conservatively by chemotherapy, hormonal therapy, and/or irradiation according to the diagnosis. Periacetabular lesions (Enneking zone 2) can be treated in the same way if they are osteoblastic or osteolytic with a small size and a positive reaction to irradiation is expected (breast, thyroid, prostate, myeloma, and lymphoma). Weight-bearing is strictly forbidden during the radiation therapy to reduce the risk for an iatrogenic fracture [24]. The response to treatment and the control of pain should be carefully evaluated at follow-up. In the case of pathological fracture, persistence of pain for two months after completion of treatment [25], or radiological signs of local progression, the patients should be referred to an orthopedic surgeon for surgical treatment since they are now in either Class 2 or Class 3.

## 9. Minimally Invasive Treatment

Patients evaluated as Class 4 do not benefit from a surgical resection of the metastatic lesion. They are treated conservatively trying to improve quality of life. Radiation therapy is quite effective in providing relief from painful bone metastasis: 50–80% of patients experience improvement in their pain and 20–50% of the treated patients have even complete pain relief [26, 27]. So the external irradiation is the standard care for patients with localized bone pain and results in the palliation of the majority of these patients. However, some patients do not experience any pain relief. Furthermore, patients who have recurrent pain at a site previously irradiated may not be eligible for further radiation therapy secondary to limitations in normal tissue tolerance. Image-guided percutaneous methods of tumor destruction have rapidly evolved for benign skeletal lesions and more recently for palliation of painful bone metastasis. Because of shortcomings of the currently available therapies for painful metastatic disease, there is a need for alternative treatment strategies. All these new techniques are based on the use of percutaneous image-guided methods to deliver tissue ablative materials or devices inside the metastatic lesion. In the literature described procedures are the local application of ethanol, laser-induced interstitial thermotherapy, cryoablation, and radiofrequency ablation. Additionally a new and promising technology is the electrochemotherapy, but it is still under investigation for the efficacy in bone metastasis and the clinical application [28, 29]. These percutaneous treatments should be considered if the patient has pain not controllable by narcotic analgesics or not responding to earlier applied therapies and is not eligible for a surgical resection. The device based ablation methods may also be combined with the use of methyl methacrylate for further stabilization.

### 9.1. Ethanol and Thermotherapy

The easiest and presumably the most cost-effective treatment is the injection of ethanol (95%) under CT guidance [30]. The rare existing reported results in the literature indicate a complete pain relief in 16% and no effect in 28% of the patients [30]. For the laser-induced thermotherapy only 3 cases are reported with a pain relief of maximal 45% [31].

### 9.2. Cryoablation

Cryoablation has a long history of successful treatment of neoplasms in several organs, especially in the prostate. The rapid freezing adjacent to the probe results in intracellular ice formation and with more distance to the probe the cooling causes osmotic differences. Both of these cellular changes induce cell death. Available preliminary data suggest that cryoablation is effective in treating painful secondary bone neoplasms [32, 33].

### 9.3. Radiofrequency Ablation

Radiofrequency ablation utilizes a high-frequency alternating current that is passed from the needle electrode into the surrounding tissue, resulting in frictional heating and necrosis. Dupuy [34] first reported that radiofrequency ablation of metastases involving the bone may provide pain relief. The first results were engaging and a multicenter study was performed [35] to obtain reliable data: 95% of the included patients experienced a clinical significant pain reduction during the observed time.

### 9.4. Promising New Technique: Electrochemotherapy

Electrochemotherapy (ECT) is the combined effect of electric fields and chemotherapeutics to treat tumor [29]. Using electric pulses bleomycin can enter the tumor cells and accumulates intracellularly. A local effect is created without any unspecific toxicity to normal tissue. Clinical application of ECT mainly focuses on cutaneous and subcutaneous metastatic tumor nodules. Laboratory findings in rats suggest good clinical and histological results using electrochemotherapy in bone metastasis [28]. No important side effects were observed and it seems that in contrary to the other ablation techniques the mechanical bone strength is preserved. Further clinical investigations have to be performed to evaluate this promising procedure.

### 9.5. Acetabuloplasty

**Figure 7:**
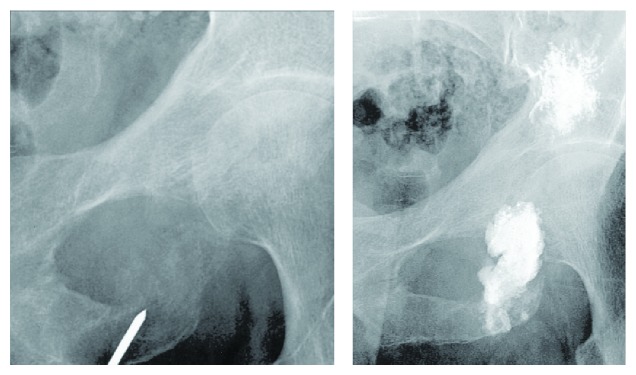
Intra- and postoperative radiographies of an acetabuloplasty.

Cotten et al. [36] adapted the vertebroplasty technique and applied the same principles for the management of secondary osteolytic lesions around the acetabulum. Acetabuloplasty consists of a percutaneous injection of low viscosity acrylic cement into the osteolytic cavity (Figure 7).

The principal goal is an immediate improvement of the mechanical properties of the affected bone, especially a higher resistance to compressive stresses lowering the fracture risk [37]. Additionally the exothermic reaction during the polymerization of the cements exerts a local cytotoxic reaction against the tumor. Complete pain relief is achieved in 59% of patients [38]. The combination between an ablation therapy and cementoplasty seems to boost the overall effect. A 100% success rate concerning pain control was reported for radiofrequency ablation together with cementoplasty at the level of the spine [39].

Generally very low complication rates are observed using the percutaneous procedures. Due to the minimal skin incision infection risk is very low. Rare cases of cement protrusion in the hip joint are described with no significant functional loss [40, 42]. Ablation methods as radiofrequency or cryotherapy are contraindicated if the tumor is closer than 1 cm to important structures, for example, spinal cord, major nerves, vessels, or intestine. A short overview for the reported results of minimally invasive techniques is shown in Table 6.

## 10. Conclusion

Pelvic bone metastases are a growing concern in the field of orthopedic surgery. Every patient needs careful evaluation and staging. Wide resection results in an improved survival only in solitary metastasis with favorable prognosis. Osteolytic lesions in the periacetabular regions can lead to pathological fractures and important functional impairment. Different reconstruction techniques for the acetabulum are available; the choice depends on the patient's prognosis, size of the bone defect, and response of the tumor to adjuvant treatment. Osteosclerotic acetabular lesions and lesions in the iliac wing and the anterior pelvis are commonly treated conservatively with external irradiation to reduce pain and as local control. If all the conservative treatments are exhausted and the patient is not eligible for surgery, a percutaneous ablation therapy with radiofrequency or cryoablation can be considered. Cementoplasty is another minimal invasive solution to reduce pain and additionally to reinforce the residual bone.

Every patient needs his individual treatment of the metastasis to provide the best possible life quality despite the advanced stage of disease. A good organized collaboration between the different specialists as oncologists, radiation therapist, and orthopedic surgeon is mandatory. The use of the presented classifications and algorithms simplifies the teamwork and helps to avoid under- or overtreatment of pelvic bone metastasis.

## Figures and Tables

**Table 1:** Characteristics of the different patient classes comparing metastatic lesions in long bones and pelvis.

Class	Long bones	Pelvis
1	Solitary metastatic lesion	
	Primary with good prognosis	
	(well-differentiated thyroid, prostate, breast sensitive to adjuvants, rectum, clear-cell renal, lymphoma, and myeloma)	
	Interval over three years since detection of the primary	

2	Pathological fracture at any site	Pathological fracture in periacetabular region

3	Impending fracture in a major weight bearing bone	Supra-acetabular osteolytic lesion

4	Multiple osteoblastic lesions at all sites	Multiple osteoblastic lesions at all sites
	Osteolytic or mixed lesions in nonstructural bone	Osteolytic or mixed lesions in iliac wing and anterior pelvis
	Osteolytic lesion with no impending fracture in major weight bearing bone	Small periacetabular osteolytic lesions

**Table 2:** Summary of surgical techniques for pelvic metastasis.

Patient	Site of lesion	Resection	Reconstruction
Class 1	Zones 1, 3	Wide margins	None
	Zone 2	Wide margins	Harrington procedure Megaprosthesis Saddle prosthesis Massive allograft with THR

			**Harrington I defect**
			Curettage, cement
			Conventional THR
			**Harrington II defect**
			THR with reinforcement ring
			**Harrington III defect**
Classes 2, 3	Zone 2	Marginal, intralesional	Harrington procedure
			Defect filling with cement or
			allograft and THR
			**Harrington IV defect**
			Megaprosthesis
			Saddle prosthesis
			Massive allograft with
			THR

THR: total hip replacement.

**Table 3:** Scoring system and recommended treatment for pelvic metastasis in patients of classes 2 and 3.

Survival	Site of defect	Size of defect	Response to adjuvant therapy
<1 year = 1	Periacetabular = 1	Small supra-acetabular or medial wall = 2	Yes = 0
1-2 years = 2		Medial and lateral wall = 4	No = 3
>2 years = 3		Protrusio acetabuli = 6	

Up to 5 points:	curettage or conventional total hip replacement		
5 to 10 points:	complex total hip replacement (reinforcement ring, Harrington procedure)		
10 to 13 points:	megaprosthesis, saddle prosthesis, and massive allograft		

**Table 4:** Predictive survival and scoring for the protocol.

Survival	Source of metastasis
<1 year (1 point)	Unknown
	Melanoma
	Lung
	Pancreas
	Thyroid (undifferentiated)
	Stomach

1 to 2 years (2 points)	Colon
	Breast (not responding to adjuvants)
	Liver
	Uterus (responding to adjuvants)

Over 2 years (3 points)	Thyroid (differentiated)
	Myeloma
	Lymphoma
	Breast (responding to adjuvants)
	Rectum
	Prostate
	Kidney

**Table 5:** Predictive response to adjuvant treatment and scoring for the protocol.

Responsive to adjuvant therapy (0 points)	Breast
	Thyroid
	Myeloma
	Lymphoma
	Prostate

Nonresponsive to adjuvant therapy (3 points)	Kidney
	Gastrointestinal tumor
	Lung
	Uterus
	Pancreas

**Table 6:** Overview for reported results in minimally invasive techniques.

Technique	Author	Year	Patients	Follow-up	Complications	Effect
Ethanol therapy	Gangi et al. [30]	1994	25	2 weeks	none	Complete pain relief in 16%
						Partial pain relief in 75%

Laser-induced thermotherapy	Groenemeyer et al. [31]	2002	3	3 months	none	45% pain reduction

Cryoablation	Callstrom et al. [33]	2006	14	6 months	none	Pain relief in 100%

Radiofrequency ablation	Goetz et al. [35]	2004	43	16 weeks	1 skin burn	Pain relief in 95%
					1 transient bowel and bladder	
					incontinence (metastasis of sacrum)	
					1 fracture of acetabulum	

Acetabuloplasty	Cotten et al. [36]	1995	18	7 months	Recurrent pain	Pain relief in 81%
					fever/inflammatory processes	
	Marcy et al. [40]	2000	18	4.6 months	1 acetabular fracture	Pain relief in 81%
	Hierholzer et al. [41]	2003	5	—	None	Pain relief in 100%
	Kelekis et al. [42]	2005	14	—	1 intraarticular leakage	Pain relief in 92%
					1 leakage near pudendal nerve	
	Maccauro et al. [38]	2008	25	6 months	2 venous injection of cement	Complete pain relief in 59%
						Partial pain relief in 49%
